# Bibliometric analysis of global scientific research on carbapenem resistance (1986–2015)

**DOI:** 10.1186/s12941-016-0169-6

**Published:** 2016-09-23

**Authors:** Waleed M. Sweileh, Naser Y. Shraim, Samah W. Al-Jabi, Ansam F. Sawalha, Adham S. AbuTaha, Sa’ed H. Zyoud

**Affiliations:** 1 Department of Physiology, Pharmacology and Toxicology, College of Medicine and Health Sciences, An-Najah National University, Nablus, 44839 State of Palestine; 2 Department of Pharmaceutical Chemistry and Technology, College of Medicine and Health Sciences, An-Najah National University, Nablus, 44839 State of Palestine; 3 Department of Clinical and Community Pharmacy, College of Medicine and Health Sciences, An-Najah National University, Nablus, 44839 State of Palestine

**Keywords:** Carbapenem, Resistance, Bibliometric analysis

## Abstract

**Background:**

Antimicrobial resistance is a global public health challenge and carbapenem resistance, in particular, is considered an urgent global health threat. This study was carried out to give a bibliometric overview of literature on carbapenem resistance. In specific, number of publications, top productive countries and institutes, highly cited articles, citation analysis, co-authorships, international collaboration, top active authors, and journals publishing articles on carbapenem resistance were analyzed and discussed.

**Methods:**

Specific keywords pertaining to carbapenem resistance were used in Scopus database. Quantitative and qualitative analysis of retrieved data were presented using appropriate bibliometric indicators and visualization maps.

**Results:**

A total of 2617 journal articles were retrieved. The average number of citations per article was of 21.47. The growth of publications showed a dramatic increase from 2008 to 2015. Approximately 9 % of retrieved articles on carbapenem resistance were published in *Antimicrobial Agents and Chemotherapy* journal. Retrieved articles were published by 102 different countries. The United States of America (USA) contributed most with 437 (16.70 %) articles followed by China with 257 (9.82 %) articles. When productivity was stratified by population size, Greece ranked first followed by France. Greece also ranked first when data were stratified by gross domestic product (GDP). Asian countries have lesser international collaboration compared with other countries in the top ten list. Five of top ten productive institutes were Europeans (France, the UK, Greece, Italy, and Switzerland) and two were Asians (China and South Korea). Other active institutes included an Israeli and a Brazilian institute. Four of the top ten cited articles were published in *Antimicrobial Agents and Chemotherapy* journal and two were published in *The Lancet Infectious Diseases*.

**Conclusion:**

There was a dramatic increase in number of publications on carbapenem resistance in the past few years. These publications were produced from different world regions including Asia, Europe, Middle East, and Latin America. International collaboration needs to be encouraged particularly for researchers in Asia. Molecular biology and epidemiology dominated the theme of the top ten cited articles on carbapenem resistance. This bibliometric study will hopefully help health policy makers in planning future research and allocating funds pertaining to carbapenem resistance.

## Background

In 2014, the World Health Organization (WHO) issued a report on antimicrobial resistance (AMR) stating that AMR is becoming a global challenge that threatens the clinical benefit of many important antimicrobial agents [1]. Of a real concern in the WHO report was a warning about spread of resistance among different Gram-negative bacteria to carbapenems. This created a great deal of concern among clinicians, microbiologists and pharmacologists because carbapenems are considered antibiotics of last resort in combating serious infections [2]. Carbapenems are β-lactam antibiotics that were developed from thienamycin, which served as the parent model for other carbapenems including imipenem, ertapenem, doripenem and meropenem [3, 4]. Carbapenem resistance is a global public health challenge and efforts to minimize the spread of carbapenem resistance and risk of serious outbreaks is considered top priority [5, 6]. International health organizations, governments, healthcare providers, and researchers need to coordinate efforts and respond to this new global public health challenge by implementing programs to rationalize use of antibiotics [7–9]. As a baseline information, it is important to analyze research output published globally on carbapenem resistance. Such baseline data are needed in order to understand the current research situation and plan future research agenda accordingly.

An important and common method used to assess research activity on a certain topic is bibliometric analysis which is defined as the use of mathematical methods to analyze published articles in terms of quantity and quality [10, 11]. In bibliometric analysis, information regarding growth of publications, international collaboration, top active countries, institutes, and authors are presented. Moreover, journals publishing on the topic of interest are also presented. In some bibliometric studies, maps are presented as a method of visualization of bibliometric indicators. The retrieval of published articles on a certain topic is the first step in evaluation of literature regarding a certain problem or topic and in building evidence based clinical decisions. Retrieval of published articles on a certain topic can be achieved through the use of databases like PubMed, Google Scholar, Scopus, or Web of Science. In the past decade, a number of bibliometric studies on specific types of infections and on specific types of medical subjects like microbiology were published [12–14]. However, no bibliometric studies have been carried out on carbapenem resistance. Therefore, this study was carried out to analyze and present bibliometric indicators pertaining to literature on carbapenem resistance.

## Methods

Data collection for this study was carried out using Scopus database. The methodology used was similar to that described in previously published bibliometric studies [15–21]. Keywords used for data extraction were obtained from published review articles on carbapenem resistance. Search query used for data extraction from Scopus looked like this:

(TITLE(“carbapenem resist*” OR “imipenem resist*” OR “meropenem resist*” OR “ertapenem resist*” OR “doripenem resist*” OR “carbapenemase produ*” OR “carbapenem non-susceptible” OR “carbapenem hdroly*ing”) AND PUBYEAR < 2016) OR ((TITLE-ABS-KEY(Ndm-1 OR “New Delhi metallo*” OR “carbapenemase” OR “extended-spectrum *lactamase*”) AND TITLE-ABS(“carbapenem resist*”)) OR ((TITLE (“coli” OR “enterobacter*” OR “aeruginosa” OR “baumannii” OR “klebsiella”)) AND KEY(“carbapenem resist*”)) AND PUBYEAR < 2016) AND (LIMIT-TO(SRCTYPE,”j”)) AND (EXCLUDE(DOCTYPE,”er”))

The asterisk was used for certain words to retrieve all potential correct words while the quotation marks were used to retrieve correct and exact phrases. Validity of the search query was confirmed by manual analysis of the top 100 cited articles. In this study, manual analysis of the top 100 cited articles showed no deviation from required goal of retrieving articles on carbapenem resistance. The time span for data collection was all previous years until 2015. Retrieved documents were refined and limited to journal articles. Errata and undefined documents were excluded. Bibliometric indicators were presented as top ten productive countries, institutes, authors, journals, and highly cited articles. For evaluation of quality of publications, *h*-index, average number of citations per article, and total number of citations were used [13, 22–25]. For quality of journals publishing articles on carbapenem resistance, impact factor (IF) and scientific journal rankings (SJR) were presented for each journal. Impact factors for journals were obtained from the latest Journal Citation Report published by Thompson Reuters [26] while SJR for journals were obtained from Scimago Journal Rank [27]. Poisson loglinear regression was carried out using the annual worldwide productivity as a dependent variable. Predictor independent variables were used as covariates in the model. The covariates were: number of publications produced annually by top three countries, number of articles with the keyword “hospital”, and number of articles produced annually in the field of molecular biology/microbiology. Author co-citation analysis (ACA) was presented as density visualization map using VOSviewer techniques [28].

## Results

**Fig. 1:**
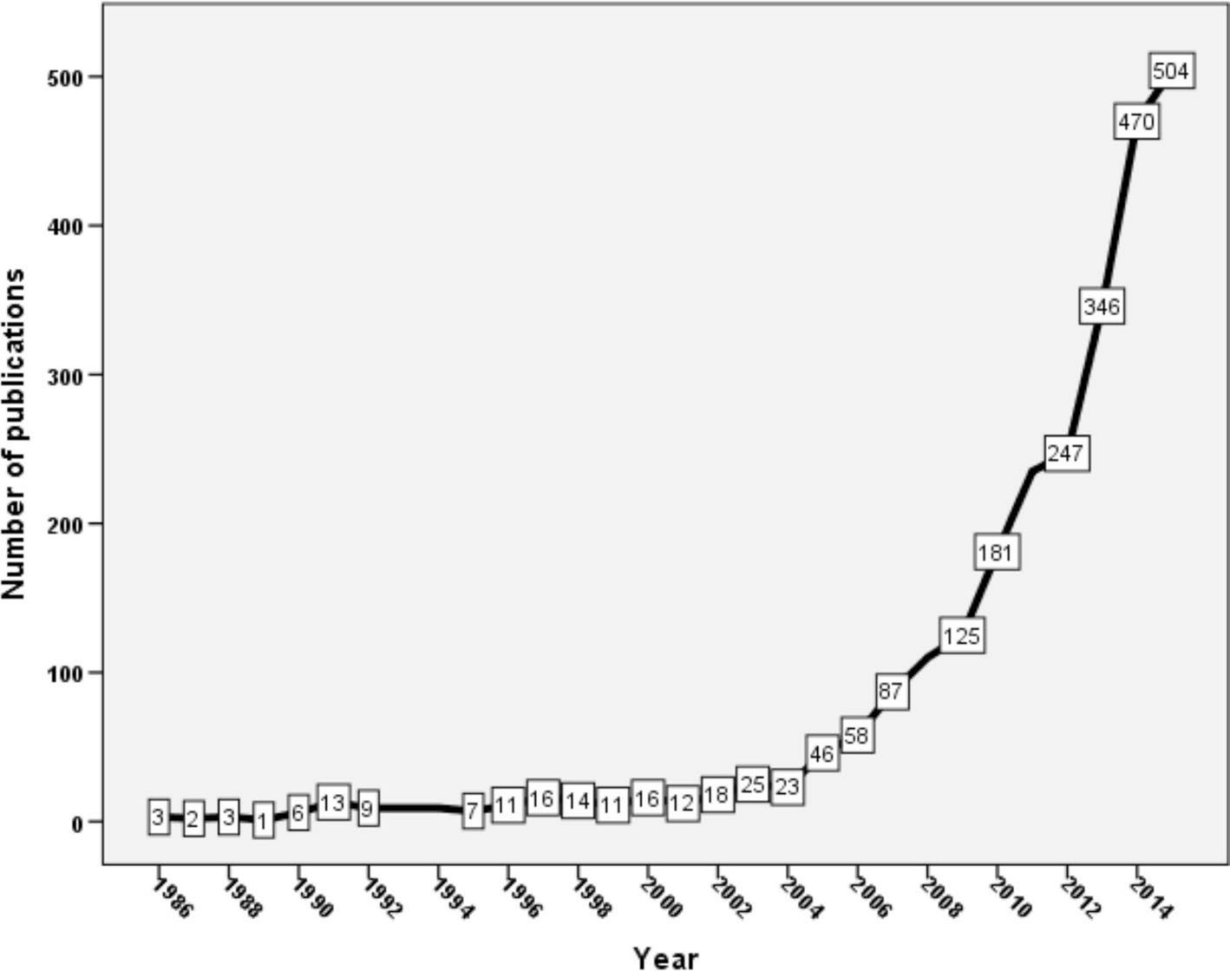
Temporal distribution of production of publications about carbapenem resistance (1986–2015)

A total of 2617 articles were retrieved. The average number of citations per article was 21.47. The *h*-index of retrieved articles was 102. Most retrieved documents were original articles (2126, 81.24 %) and most of these documents were written in English language (2355, 89.99 %). Chinese language ranked second with 120 (4.59 %) articles. Growth of publications on carbapenem resistance started in 1986 and remained at an average of ten articles per year until late 1990s. Growth of publications increased slowly from 2000 to 2008 followed by a dramatic rise in the number of publications (Fig. 1). The oldest articles on carbapenem resistance were published in 1986. These old articles discussed imipenem resistance in *Pseudomonas aeruginosa* and *Bacteroides fragilis* [29–31].

**Fig. 2:**
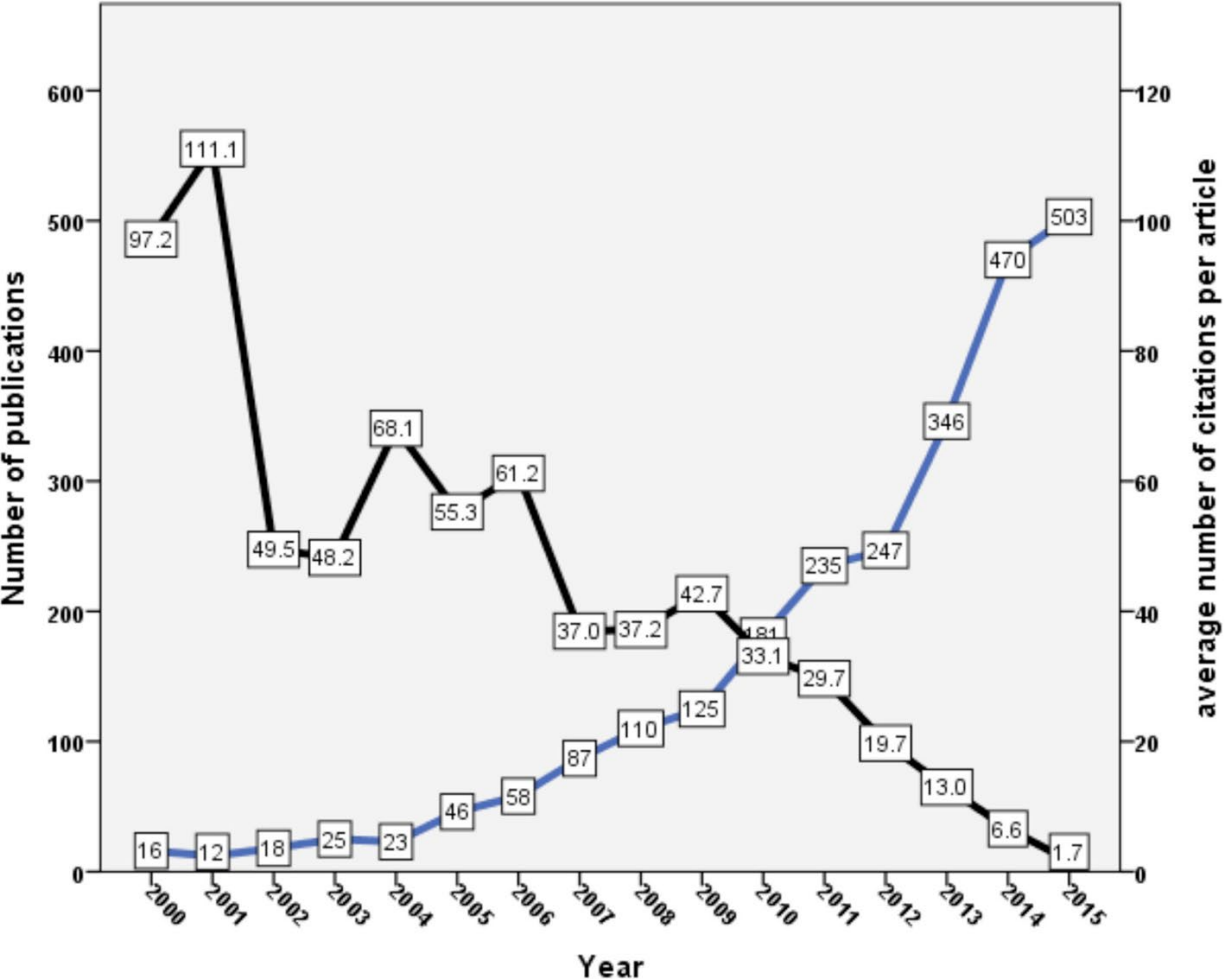
Growth of publications and citations for carbapenem resistance (1986–2015)

There was a linear increase in number of publications with time. However, the number of citations per article per year showed an inverse linear relationship with time indicating that older articles were being continuously cited with time (Fig. 2).

Relevant terms to carbapenem resistance were analyzed for number of occurrences in article titles using VOSviewer technique. Analysis showed that “*pneumoniae*” was the most frequent term (305 occurrences) followed by “*Acinetobacter baumannii*” (302 occurrences), “*Pseudomonas aeruginosa*” (132 occurrences), “resistant *Enterobacteriaceae*” (124 occurrences), and “metallo β lactamase” (21 occurrences).

Core journals that published most on carbapenem resistance were shown in Table 1. A total of 1078 (41 %) articles on carbapenem resistance were published by top ten journals listed in Table 1. Approximately 9 % (n = 232) of retrieved articles on carbapenem resistance were published in *Antimicrobial Agents and Chemotherapy* journal (IF of 4.476). Journals in the top ten list were in the field of antimicrobials, microbiology, infection, and antimicrobial resistance. Furthermore, all journals in the top ten list were prestigious and influential ones. The journal with the highest IF was *Journal of Antimicrobial Chemotherapy* (IF = 5.313).

**Table 1:** Top ten journals publishing on carbapenem resistance (1986–2015)

SCR	Journal	Number of articles (%)	Total number of citations (R)	Number of citations per article (R)	*h*-index (R)	IF (R)	SJR (R)
1st	*Antimicrobial Agents and Chemotherapy* (USA)	232 (8.87)	12,136 (1)	52.31 (1)	57 (1)	4.476 (3)	2.01 (2)
2nd	*Journal of Antimicrobial Chemotherapy* (USA)	201 (7.68)	7245 (2)	36.04 (3)	51 (2)	5.313 (1)	1.98 (3)
3rd	*Journal of Clinical Microbiology* (USA)	119 (4.55)	4502 (3)	22.40 (6)	39 (3)	4.232 (5)	1.89 (4)
4th	*International Journal of Antimicrobial Agents* (Netherlands)	101 (3.86)	2777 (5)	23.34 (5)	27 (6)	4.269 (4)	1.28 (6)
5th	*Clinical Microbiology and Infection* (UK)	85 (3.04)	3079 (4)	37.55 (2)	30 (4)	5.197 (2)	2.29 (1)
6th	*Infection Control and Hospital Epidemiology* (USA)	84 (3.21)	2403 (6)	23.79 (4)	28 (5)	4.175 (6)	1.80 (5)
7th	*Diagnostic Microbiology and Infectious Disease* (USA)	82 (3.13)	1163 (7)	13.85 (8)	20 (7)	2.457 (9)	1.02 (8)
8th	*Journal of Medical Microbiology* (UK)	66 (2.52)	956 (8)	11.25 (9)	19 (8)	2.248 (10)	0.91 (9)
9th	*Microbial Drug Resistance* (USA)	56 (2.14)	642 (10)	9.73 (10)	15 (10)	2.490 (8)	0.91 (9)
10th	*Journal of Hospital Infection* (UK)	52 (1.99)	937 (9)	16.73 (7)	16 (9)	2.544 (7)	1.05 (7)

*SCR* standard competition ranking, *SJR* scientific journal rank, *IF* impact factor, *R* rank

Retrieved articles were published by 102 different countries distributed in all world regions. However, 68.44 % (n = 1791) of retrieved articles were published by top ten ranking countries. The list of top ten productive countries included five countries in Europe, three in Asia, one in Northern America, and one in Latin America (Table 2). The United States of America (USA) had the greatest contribution with 437 (16.70 %) articles followed by China with 257 (9.82 %) articles. When productivity was stratified by population size, Greece ranked first followed by France. However, when productivity was stratified by gross domestic product (GDP), Greece ranked first followed by Spain. When quality of publications was assessed for each country by calculating the average number of citations per article, the United Kingdom (UK) ranked first followed by Greece and France. International collaboration, assessed by the number of articles with multiple country affiliations, showed that UK had the highest percentage of articles with international collaboration (82; 54.92 %) followed by France (117; 53.92 %) and Greece (40; 38.46 %). Top three countries (UK, France and Greece) engaged in international collaboration were the same top three countries which had the highest average number of citations per article.

**Table 2:** Top ten productive countries on carbapenem publications (1986–2015)

SCR	Country	Articles N = 2469 (%)	Articles/10 million inhabitants (rank)	Articles/trillion GDP (rank)	Total citation (rank)	Citations/article (rank)	*h*-index (rank)	Number of collaborating countries	Number (%)^a^ of documents with international authors
1st	United States	437 (16.70)	13.70 (7)	25.09 (9)	14,667 (1)	33.56 (4)	62 (1)	39	145 (33.18)
2nd	China	257 (9.82)	1.88 (9)	24.83 (10)	2069 (9)	8.05 (10)	23 (7)	16	29 (11.28)
3rd	France	217 (8.29)	32.77 (2)	76.71 (3)	7835(3)	36.11 (3)	45 (3)	55	117 (53.92)
4th	United Kingdom	151 (5.77)	23.39 (5)	50.52 (8)	8909 (2)	59.00 (1)	49 (2)	57	82 (54.92)
5th	Italy	146 (5.58)	24.02 (4)	68.19 (5)	3900 (4)	26.71 (7)	31 (5)	43	44 (30.14)
6th	India	137 (5.24)	1.06 (10)	66.86 (6)	2407 (8)	17.57 (8)	19 (10)	21	16 (11.68)
7th	Brazil	125 (4.78)	6.07 (8)	51.72 (7)	1781 (10)	14.25 (9)	21 (9)	10	22 (17.60)
8th	Spain	118 (4.51)	25.39 (3)	85.45 (2)	3192 (6)	27.05 (6)	31 (5)	36	24 (20.34)
9th	Greece	104 (3.97)	95.68 (1)	441.43 (1)	3778 (5)	36.33 (2)	37 (4)	39	40 (38.46)
10th	South Korea	99 (3.78)	19.55 (6)	70.21 (4)	2896 (7)	29.25 (5)	23 (7)	15	12 (12.12)

*SCR* standard competition ranking, *GDP* gross domestic product

^a^Percentage of documents with international authors was calculated by dividing number of documents with international authors by the total number of documents retrieved for the country assigned

Using Poisson loglinear regression, we examined variations of three factors on the annual number of publications on carbapenem resistance. The variables tested were (1) “hospital” as a keyword in the article; (2) subject area in the field of molecular biology/microbiology; and (3) annual productivity of top three countries. Variables were entered in Poisson regression model as continuous covariates. Table 3 shows that molecular biology/microbiology subject area (*p* < 0.01) and presence of article keyword “hospital” (*p* < 0.01) were significant predictors of worldwide research productivity on carbapenem resistance. The results indicated that the worldwide productivity will be 1.002 times greater for each extra article published in the field of molecular biology/microbiology. Similarly, the worldwide productivity will be 1.031 times greater for each extra article published with a keyword “hospital”. The annual productivity of the top three countries was not a significant predictor (*p* = 0.296) of worldwide research productivity on carbapenem resistance.

**Table 3:** Poisson loglinear regression model for predicting worldwide research productivity on carbapenem resistance

Parameter	B	*P*	Exp(B)	95 % Wald confidence interval for exp(B)	
				Lower	Upper
(Intercept)	3.058	0.000	21.277	19.621	23.072
Productivity by top three countries	−0.001	0.296	0.999	0.996	1.001
Hospital	0.030	0.000	1.031	1.027	1.034
Molecular	0.004	0.001	1.004	1.002	1.006

Dependent variable: worldwide productivity. Model: Hospital, Molecular, and productivity by top three countries

*B* coefficient estimates, *Exp(B)* exponentiated values of the coefficients

Research and academic institutes involved in carbapenem resistance were presented as top ten productive institutes in Table 4. Five of top ten productive institutes were in Europe (France, Greece, UK, Italy and Switzerland) and two were in Asia (China and South Korea). Other active institutes were ones in the USA, Israel and Brazil. When ranked based on the number of published articles, *Hopital de Bicetre* in France ranked first followed by the *University of Athens Medical School* in Greece*, Zhejiang University* in China, *VA medical center* in the USA and *Health Protection Agency* in the UK. However, when top ten productive institutes were ranked based on quality of publications assessed by average number of citations per article, *Health Protection Agency* in the UK ranked first followed by *Centers for Disease Prevention and Control* (CDC) in the USA. At least three of top ten institutes were governmental health research institutes (*VA Medical Center, CDC and Health Protection Agency*).

**Table 4:** Top ten list of institutes publishing on carbapenem resistance (1896–2015)

SCR^a^	Institution	Number of documents N = 2617 (%)	Total citation (rank)	Citations/article (rank)	*h*-index (rank)	Affiliation country
1st	Hopital de Bicetre	53 (2.03)	3254 (2)	61.40 (3)	25 (2)	France
2nd	University of Athens Medical School	45 (1.72)	1917 (4)	42.60 (6)	23 (3)	Greece
3rd	Zhejiang University	43 (1.64)	1105 (7)	25.70 (8)	18 (7)	China
4th	VA Medical Center	39 (1.49)	965 (8)	24.74 (9)	18 (7)	United States
5th	Health Protection Agency	35 (1.33)	4392 (1)	125.49 (1)	26 (1)	United Kingdom
6th	Tel Aviv Sourasky Medical Center	34 (1.30)	1900 (5)	55.88 (4)	22 (4)	Israel
7th	Universita degli Studi di Siena	33 (1.26)	1792 (6)	54.30 (5)	19 (5)	Italy
8th	Centers for Disease Control and Prevention	32 (1.22)	2238 (3)	69.94 (2)	19 (5)	United States
9th	Yonsei University College of Medicine	31 (1.18)	803 (9)	25.90 (7)	15 (9)	South Korea
10th	Universidade Federal de Sao Paulo	29 (1.11)	620 (10)	21.38 (10)	11 (11)	Brazil
10th	Universite de Fribourg	29 (1.11)	452 (11)	15.59 (11)	13 (10)	Switzerland

*SCR* standard competition ranking

^a^Equal institutes have the same ranking number

Active researchers in the field of carbapenem resistance are shown in Table 5 as top ten active authors. Professor Nordmann ranked first in number and quality of publications with 95 publications and *h*-index of 36. Professor Poirel ranked second in number of publications with 71 publications and *h*-index of 32 while Carmeli ranked third with 47 publications and *h*-index of 26. Both Nordman and Poirel are affiliated with the same institute (Inserm U914, Kremlin-Bicêtre, France and Département de médecine, Faculté des sciences, Université de Fribourg, Fribourg, Switzerland). Both professors have extensive research collaboration. Same applies to Professors Carmeli and Navon-Venezia who share the same affiliation and have a noticeable research collaboration. Six of top ten active authors on carbapenem resistance were in Europe, two in Israel, one in the USA and one in South Korea.

**Table 5:** Top ten authors publishing on carbapenem resistance (1986–2015)

SCR^a^	Author	Number of published articles	Color (number of cluster)	Total citation (R)	*h*-index (R)	Country
1st	Nordmann	95	Blue (3)	5545 (1)	36 (1)	Switzerland/France
2nd	Poirel	71	Blue (3)	4129 (4)	32 (3)	Switzerland/France
3rd	Carmeli	47	Green (2)	2449 (5)	26 (5)	Israel
4th	Woodford	44	Red (1)	5083 (3)	32 (3)	United Kingdom
5th	Livermore	40	Red (1)	5420 (2)	33 (2)	United Kingdom
6th	Tsakris	36	Red (1)	1075 (9)	18 (8)	Greece
7th	Bonomo	35	Green (2)	1038 (10)	18 (8)	United States
8th	Rossolini	35	Red (1)	2010 (6)	19 (7)	Italy
9th	Lee	35	Red (1)	1708 (7)	17 (10)	South Korea
10th	Navon-Venezia	30	Green (2)	1533 (8)	21 (6)	Israel

*R* rank, *SCR* standard competition ranking

^a^Equal authors have the same ranking number, and then a gap is left in the ranking numbers

**Fig. 3:**
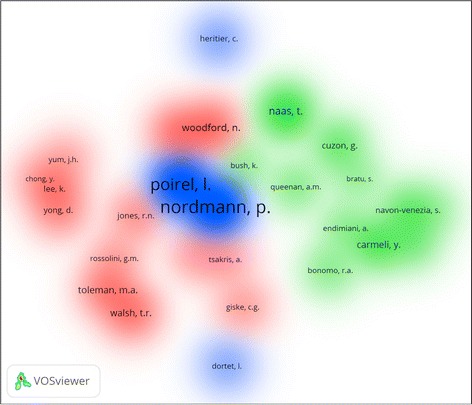
Author co-citation analysis for carbapenem resistance publications (1986–2015). Minimum of 100 citations per author, 28 authors were included

Author co-citation analysis (ACA) for top ten active authors was carried out using VOSviewer technique. The ultimate number of authors included was 28 based on a minimum number of 100 citations per author. The map produced included 28 authors distributed into three clusters (red, green, and blue) as shown in Fig. 3. Cluster number one included 13, cluster two included 11, and cluster three included four authors. Some of the authors might not be shown clearly in the map because some of the names were overlapping.

The following top active authors were present in cluster number one (red) and were commonly co-cited together: Woodford, Livermore, Tsakris and Lee. Cluster number two (green) included the following top active authors: Carmeli, Bonomo, and Navon-Venezia. Cluster number three (blue) included the following top active authors: Nordmann and Poirel.

In cluster number two (green), both Carmeli and Navon-Venezia are Israeli researchers, the former is in the field of molecular biology and the latter is in the field of preventive medicine. It is also noteworthy that both Nordman and Poirel (blue cluster) are affiliated with the same institute.

Top ten cited articles on carbapenem resistance were shown in Table 6 [32–41]. Four of the top ten cited articles were published in *Antimicrobial Agents and Chemotherapy* journal and two were published in *The Lancet Infectious Diseases*. The article that received the highest number of citations was “*Emergence of a new antibiotic resistance mechanism in India, Pakistan, and the UK: A molecular, biological, and epidemiological study*”. It was published in 2010 and received a total of 1212 citations. Carbapenem resistance in *Klebsiella pneumoniae* was mentioned in the title of five top cited articles. Professors Poirel and Nordmann had good contribution to top ten cited articles.

**Table 6:** Top ten cited articles on carbapenem resistance (1986–2015)

SCR	Authors	Title	Year	Source title	Number of citations
1st	Kumarasamy et al. [34]	Emergence of a new antibiotic resistance mechanism in India, Pakistan, and the UK: a molecular, biological, and epidemiological study	2010	*The Lancet Infectious Diseases*	1212
2nd	Yong et al. [41]	Characterization of a new metallo-β-lactamase gene, bla NDM-1, and a novel erythromycin esterase gene carried on a unique genetic structure in *Klebsiella pneumoniae* sequence type 14 from India	2009	*Antimicrobial Agents and Chemotherapy*	835
3rd	Nordmann et al. [36]	The real threat of *Klebsiella pneumoniae* carbapenemase-producing bacteria	2009	*The Lancet Infectious Diseases*	689
4th	Yigit et al. [40]	Novel carbapenem-hydrolyzing β-lactamase, KPC-1, from a carbapenem-resistant strain of *Klebsiella pneumoniae*	2001	*Antimicrobial Agents and Chemotherapy*	673
5th	Nordmann et al. [37]	Global spread of carbapenemase producing *Enterobacteriaceae*	2011	*Emerging Infectious Diseases*	643
6th	Poirel and Nordmann [38]	Carbapenem resistance in *Acinetobacter baumannii*: mechanisms and epidemiology	2006	*Clinical Microbiology and Infection*	474
7th	Woodford et al. [39]	Multiplex PCR for genes encoding prevalent OXA carbapenemases in *Acinetobacter* spp.	2006	*International Journal of Antimicrobial Agents*	427
8th	Lauretti et al. [35]	Cloning and characterization of bla(VIM), a new integron-borne metallo-β-lactamase gene from a *Pseudomonas aeruginosa* clinical isolate	1999	*Antimicrobial Agents and Chemotherapy*	395
9th	Bratu et al. [33]	Rapid spread of carbapenem-resistant *Klebsiella pneumoniae* in New York City: a new threat to our antibiotic armamentarium	2005	*Archives of Internal Medicine*	372
10th	Bradford et al. [32]	Imipenem resistance in *Klebsiella pneumoniae* is associated with the combination of ACT-1, a plasmid-mediated AmpC β-lactamase, and the loss of an outer membrane protein	1997	*Antimicrobial Agents and Chemotherapy*	358

*SCR* standard competition ranking

## Discussion

In this study, a bibliometric overview of worldwide publications on carbapenem resistance was presented. Extensive research review showed that few studies were published on bibliometrics of carbapenems. These studies were published in Chinese journals and in Chinese language. These Chinese studies were specific to a certain type of bacteria or specific to New-Delhi metallo β-lactamase gene responsible for carbapenem resistance [42–44]. Our study is the first bibliometric study published in English.

The steep rise in number of publications on carbapenem resistance reflects real concern of scientific and medical committees about this issue and reflects the fact that the problem is spreading across the world [37, 45–48]. The CDC considers *Enterobacteriaceae* carbapenem resistance as an urgent threat [49]. Despite the fact that carbapenem resistance is relatively a recent topic, the *h*-index of retrieved articles was 104 which indicates that carbapenem resistance is a very attractive and interesting topic to many readers in the scientific and clinical field. Furthermore, the fact that approximately 10 % of retrieved documents were written in non-English language is a proof of the clinical and research popularity of carbapenem resistance.

The core journals publishing articles on carbapenem resistance are well-known and prestigious ones in the field of microbiology and infection. The majority of core journals publishing on carbapenem resistance had an IF ≥4. It is also interesting to note that one of the core journals was in specific field of drug resistance. In fact, there are at least five international journals being specialized in antimicrobial resistance such as “*Microbial Drug Resistance”, “Journal of Global Antimicrobial Resistance”,* and *“Infection and Drug Resistance”.* The core journals were mainly those issued in the USA and the UK. This explains why the majority of retrieved articles on carbapenem resistance were written in English language. No doubt, that there was a tremendous effort of Chinese researchers to publish on carbapenem resistance. However, Chinese journals are still behind on the international arena.

Contribution of various countries to publications on carbapenem resistance showed that Asian countries (China, India and South Korea) had the lowest extent of international collaboration. This might explain the low number of citations per article published from these countries. A study showed that international collaboration increases the chances of citations [50, 51]. The result that Greece and France ranked top when results were stratified by GDP or population size was surprising. Greece had suffered serious outbreaks of infections that were resistant to carbapenems and that led to a series of publications on this topic [52, 53]. Similar situation happened in France [54–56].

List of top productive institutes included *Tel Aviv Sourasky Medical Center* (Israel) and *Universite de Fribourg* (Switzerland). However, neither Israel nor Switzerland were listed in the top ten productive countries. Several hospitals in Israel had suffered from serious outbreaks of infections resistant to carbapenems [57–59]. These outbreaks of infections that threatened many Israeli hospitals has led researchers in Israel to publish reports on carbapenem resistance. This also might explain why two of top ten active authors were affiliated with Israeli institutes.

Two articles in the top ten cited articles discussed the emergence of New Delhi metallo-β-lactamase (NDM) gene responsible for carbapenem resistance. This gene belongs to carbapenemase gene family and bacteria carrying this gene are referred to as superbugs because they are resistant to most antibiotics. The term “New Delhi” was given to the gene because the bacteria having this gene was initially isolated from a patient who was visiting India [41]. This gene made carbapenem resistance an alarming risk that could spread worldwide by travelers across different parts of the world [60]. The article that first characterized this gene became one of the top ten cited articles on carbapenem resistance field. Immediately after publication of this article, an epidemiological study with molecular and biological characterization of NDM-1 gene in *Enterobacteriaceae* in India, Pakistan, and the UK was published and soon became one of the top ten cited articles in the field.

Our study has the advantage of being the first to give a bibliometric overview on carbapenem resistance. We did our best to include all potential articles and to avoid false positive and false negative results. The validation method implemented by the external microbiologist did confirm accuracy of retrieved data. However, the search query itself might not be conclusive and some articles on carbapenem resistance might be missed. Furthermore, it seems that there are Chinese medical journals that not indexed in Scopus and therefore some of the Chinese articles on carbapenem resistance might be missed. Therefore, presentation of Chinese scientists in this filed might be lesser than the actual one. Some people may argue against the use of Scopus for data retrieval. However, Scopus is comprehensive, accurate, and suitable for bibliometric studies [61].

## Conclusions

There was a dramatic increase in number of publications on carbapenem resistance in the last decade suggestive of serious spread of carbapenem resistance worldwide. Publications on carbapenem resistance originated from different world regions including Asia, Europe, and Latin America. The bulk of retrieved articles were published in microbiology and in journals related to infection control. These journals have high IF suggestive of clinical importance of this topic. International collaboration is important and need to be adopted by researchers in Asian countries to increase citations and readability. Molecular biology of genes responsible for carbapenem resistance and epidemiology of this problem dominated the top ten cited articles on this topic.

## Abbreviations

AMR: antimicrobial resistance
CDC: Centers for Disease Control and Prevention
GDP: gross domestic product
*h*-index: The Hirsch Index
IF: impact factor
NDM: New Delhi metallo-β-lactamase
SCR: standard competition ranking
SJR: SCImago Journal Rank
UK: United Kingdom
USA: United States of America
WHO: World health organization
